# Enzymatic synthesis of cellulose in space: gravity is a crucial factor for building cellulose II gel structure

**DOI:** 10.1007/s10570-021-04399-0

**Published:** 2022-01-29

**Authors:** Tomohiro Kuga, Naoki Sunagawa, Kiyohiko Igarashi

**Affiliations:** 1 Department of Biomaterial Sciences, Graduate School of Agricultural and Life Sciences, The University of Tokyo, 1-1-1 Yayoi, Bunkyo-ku, Tokyo, 113-8657 Japan; 2 VTT Technical Research Centre of Finland, Tietotie 2 VTT, P. O. Box 1000, 02044 Espoo, Finland

**Keywords:** Cellulose, Cellodextrin phosphorylase, Synthesis in vitro, Microgravity

## Abstract

**Abstract:**

We previously reported in vitro synthesis of highly ordered crystalline cellulose II by reverse reaction of cellodextrin phosphorylase from the cellulolytic bacterium *Clostridium* (*Hungateiclostridium*) *thermocellum* (*Ct*CDP), but the formation mechanism of the cellulose crystals and highly ordered structure has long been unclear. Considering the specific density of cellulose versus water, the formation of crystalline and highly ordered structure in an aqueous solution should be affected by gravity. Thus, we synthesized cellulose with *Ct*CDP stable variant at the International Space Station, where sedimentation and convection due to gravity are negligible. Optical microscopic observation suggested that cellulose in space has a gel-like appearance without apparent aggregation, in contrast to cellulose synthesized on the ground. Small-angle X-ray scattering (SAXS) and wide-angle X-ray scattering (WAXS) indicated that cellulose synthesized in space has a more uniform particle distribution in the ~ 100 nm scale region than cellulose synthesized on the ground. Scanning electron microscopy (SEM) showed that both celluloses have a micrometer scale network structure, whereas a fine fiber network was constructed only under microgravity. These results indicate that gravity plays a role in cellulose II crystal sedimentation and the building of network structure, and synthesis in space could play a role in designing unique materials.

**Graphical abstract:**

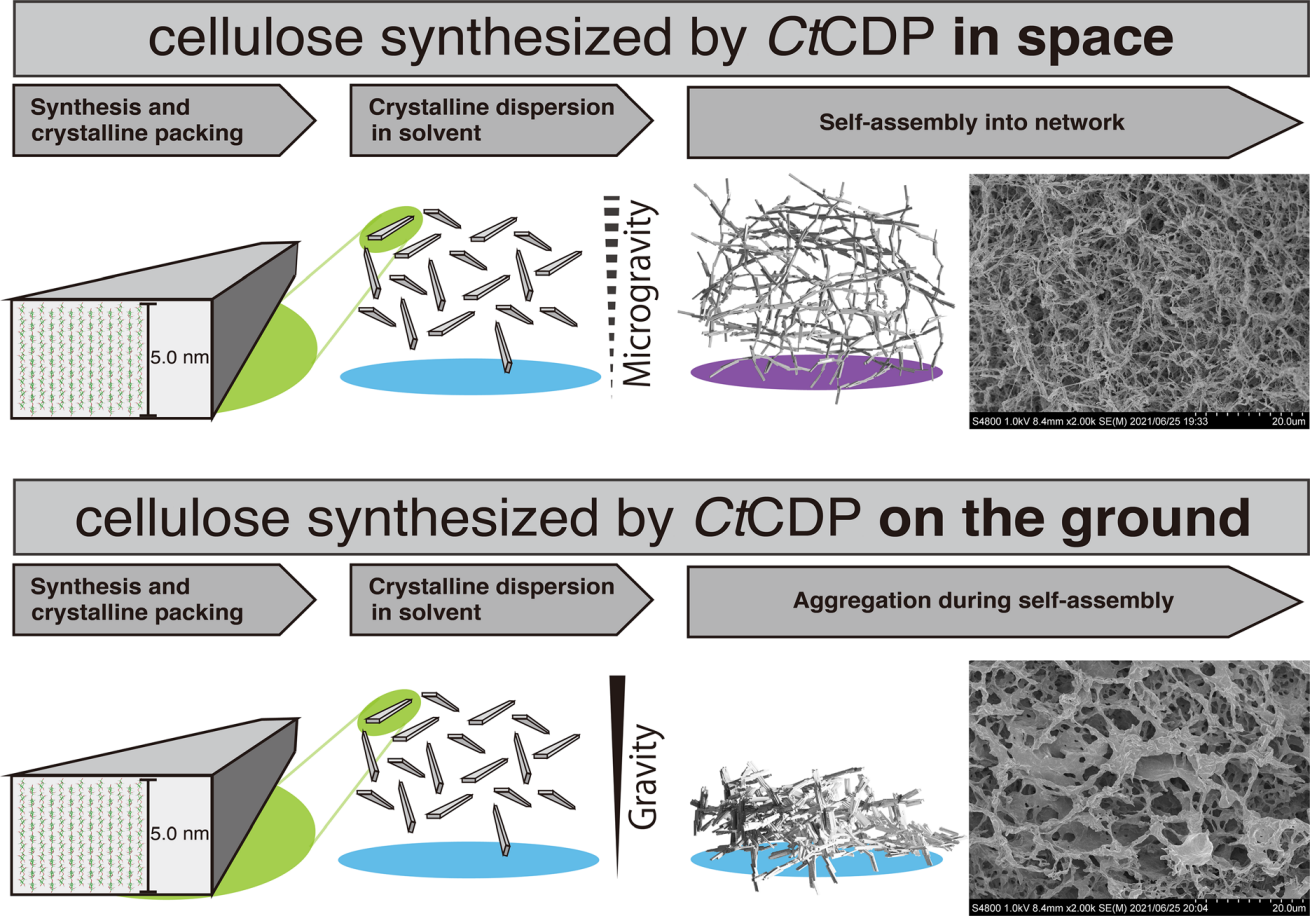

**Supplementary Information:**

The online version contains supplementary material available at 10.1007/s10570-021-04399-0.

## Introduction

Cellulose is the most abundant carbohydrate on Earth, and has been utilized by humans from ancient times. In nature, cellulose is mostly produced by woody and herbaceous plants as a cell-wall component. It is also synthesized by some microorganisms such as *Komagataeibacter xylinus* (*Acetobacter xylinum*), invertebrate animals (urochordates), or green algae (*Cladophora* species) (VanderHart and Atalla 1984; Belton et al. 1989; Larsson et al. 1997). Cellulose is a linear polymer of exclusively β-1,4-glycosidic-bonded glucose molecules synthesized by cellulose synthase complex on the cell membrane of these species. β-1,4-Glucan chains synthesized by the complex on the cell membrane spontaneously assemble and crystallize to form cellulose microfibrils (CMF; also called cellulose nanofibers, CNF). The shape of CMF depends on the geometry and morphology of the cellulose synthase complex (Brown 1996; Saxena and Brown 2005), but the mechanism of CMF formation is still unknown. Inside CMF, the β-1,4-glucan chains are bound together by hydrogen bonds and hydrophobic interaction to form a specific crystalline structure. Cellulose I_α_ and I_β_ are the smallest crystalline units of natural cellulose, and these two natural crystalline allomorphs are composed of glucan chains in parallel orientation (Atalla and VanderHart 1984; Nishiyama et al. 2002, 2003).

In contrast, cellulose II is a non-natural crystalline form originally found in mercerized and regenerated cellulose. The crystalline structure of cellulose II is significantly different from those of natural cellulose I_α_ and I_β_, having an anti-parallel orientation of cellulose molecules (Kolpak and Blackwell 1976; Langan et al. 1999; Kim et al. 2006). Cellulose II may be thermodynamically more stable, considering that it is formed in preference to metastable cellulose I_α_ or I_β_ when dissolved β-1,4-glucan chains are recrystallized.

To elucidate the formation mechanism of CMF and to develop new materials applications, the synthesis of various forms of artificial cellulose has been attempted (Uryu et al. 1983, 1985; Nakatsubo et al. 1996). Early efforts showed poor regio- and stereo-selectivity, and thus highly substrate-selective enzymatic approaches were adopted (Kobayashi et al. 1991, 2000; Kobayashi and Shoda 1995; Kobayashi 2005; Tanaka et al. 2007). Cellodextrin phosphorylase (CDP) is one of the enzymes utilized for the synthesis of cellulose in vitro. Although CDP catalyzes phosphorolysis of cellodextrin (cellooligosaccharide), it is possible to synthesize cellulose via the reverse reaction by using high concentrations of α-d-glucose-1-phosphate (α-G1P) as a glycosyl donor, with glucose and cellobiose as primary glycosyl acceptors (Alexander 1968; Sheth and Alexander 1969; Krishnareddy et al. 2002). The glycosyl donors form β-1,4-glycoside bonds with the non-reducing ends of glycosyl acceptors. In this manner, platelet lamellae of crystalline cellulose having the degree of polymerization (DP) 9 were formed in vitro (Hiraishi et al. 2009). All these studies aimed to synthesize cellulose in vitro afforded cellulose II. Pylkkänen et al*.* have found that concentrated cellulose II synthesized by CDP from *Clostridium (Hungateiclostridium) thermocellum* (*Ct*CDP) formed crystalline platelet lamellae and ribbon-like higher-ordered network structure (Pylkkänen et al. 2020). However, the mechanism of the formation of cellulose II’s supermolecular structure is still unknown, as is that of natural cellulose I_α_ and I_β_.

Protein crystallization in space enhances the quality of protein crystals due to decreased sedimentation and convection under microgravity (Vekilov 1999). This affords more orderly crystals than can be obtained on the ground, enabling researchers to obtain higher-quality X-ray diffraction data (Snell et al. 1995; Inaka et al. 2011; Nakamura et al. 2015; Tachioka et al. 2017; Yamaguchi et al. 2021). A crystal of alloy semiconductor grown on the International Space Station (ISS) also showed better quality than one grown on the ground (Inatomi et al. 2015), and an NaCl crystal grown on the ISS had different morphology from a crystal grown on Earth (Fontana et al. 2011). On the other hand, the synthesis and crystal formation of organic polymers such as cellulose under microgravity in space have not yet been investigated.

In the present study, cellulose II was synthesized in vitro using *Ct*CDP on the ISS. We investigated how gravity affects cellulose II crystalline or higher-order structure formation by comparing the product with material synthesized in the same way on the ground, employing small-angle x-ray scattering (SAXS), wide-angle X-ray scattering (WAXS), Matrix-Assisted Laser Desorption/Ionization Time-of-Flight mass spectrometer (MALDI-ToF MS) and scanning electron microscopy (SEM).

## Materials and methods

### Materials

α-G1P and pET-28b vector were purchased from Sigma-Aldrich Co. LLC (MO, US). Cellobiose and other chemical reagents were purchased from FUJIFILM Wako Pure Chemical Corporation (Osaka, Japan). Overnight Express auto-induction medium and BugBuster reagents were purchased from Merck KgaA (Darmstadt, Germany). CDP from *Clostridium (Hungateiclostridium) thermocellum* strain YM4 was initially provided by Prof. Momomitsu Kitaoka of Niigata University, Japan. *E. coli* BL21 (DE3) competent cells were purchased from Nippon Gene (Tokyo, Japan). C-Tube-LC counter-diffusion (Otálora et al. 2009) quartz capillaries were purchased from Confocal Science Inc. (Tokyo, Japan).

### Enzyme preparation

A gene coding *Δ*cys-*Ct*CDP based on CDP from *C. thermocellum* strain YM4 (GenBank: AB061316.1) was designed, in which all 11 cysteine residues were replaced with serine residues. None of the cysteine residues in *Ct*CDP are thought to form disulfide bonds (O’Neill et al. 2017). This gene was codon-optimized for expression in *E. coli* and synthesized by GenScript (NJ, US) with a 6 × His tag at the C-terminal. It was inserted into the pET-28b vector between the *Nco*I and *Xho*I sites with Ligation High (Toyobo, Osaka, Japan). The vector was transformed into *E. coli* BL21 (DE3). *Δ*cys-*Ct*CDP was expressed while transformed cells were cultivated in an Erlenmeyer flask filled with 1 L of Overnight Express auto-induction medium at 30ºC. After 18 h of cultivation, the cells were collected by centrifugation, and the crude enzyme was obtained after cell lysis with BugBuster reagents. The crude enzyme was purified on a TALON his-tag cobalt affinity column (Clontech Takara Bio USA, CA, US). The His-tagged target protein was eluted with a linear gradient of 20 mM Tris–HCl buffer pH 7.5 containing 100 mM NaCl and 500 mM imidazole. The His-tagged protein was then dialyzed against 20 mM Tris–HCl buffer with an Amicon apparatus with a 10,000 MWCO Biomax membrane filter (Merck). Anion exchange chromatography with TOYOPEARL DEAE-650S (Tosoh, Tokyo, Japan) was employed for further purification. Highly purified *Δ*cys-*Ct*CDP was eluted with a linear gradient of 20 mM Tris–HCl buffer pH 7.5 containing 250 mM NaCl and used for cellulose synthesis.

### Cellulose synthesis in vitro

**Fig. 1:**
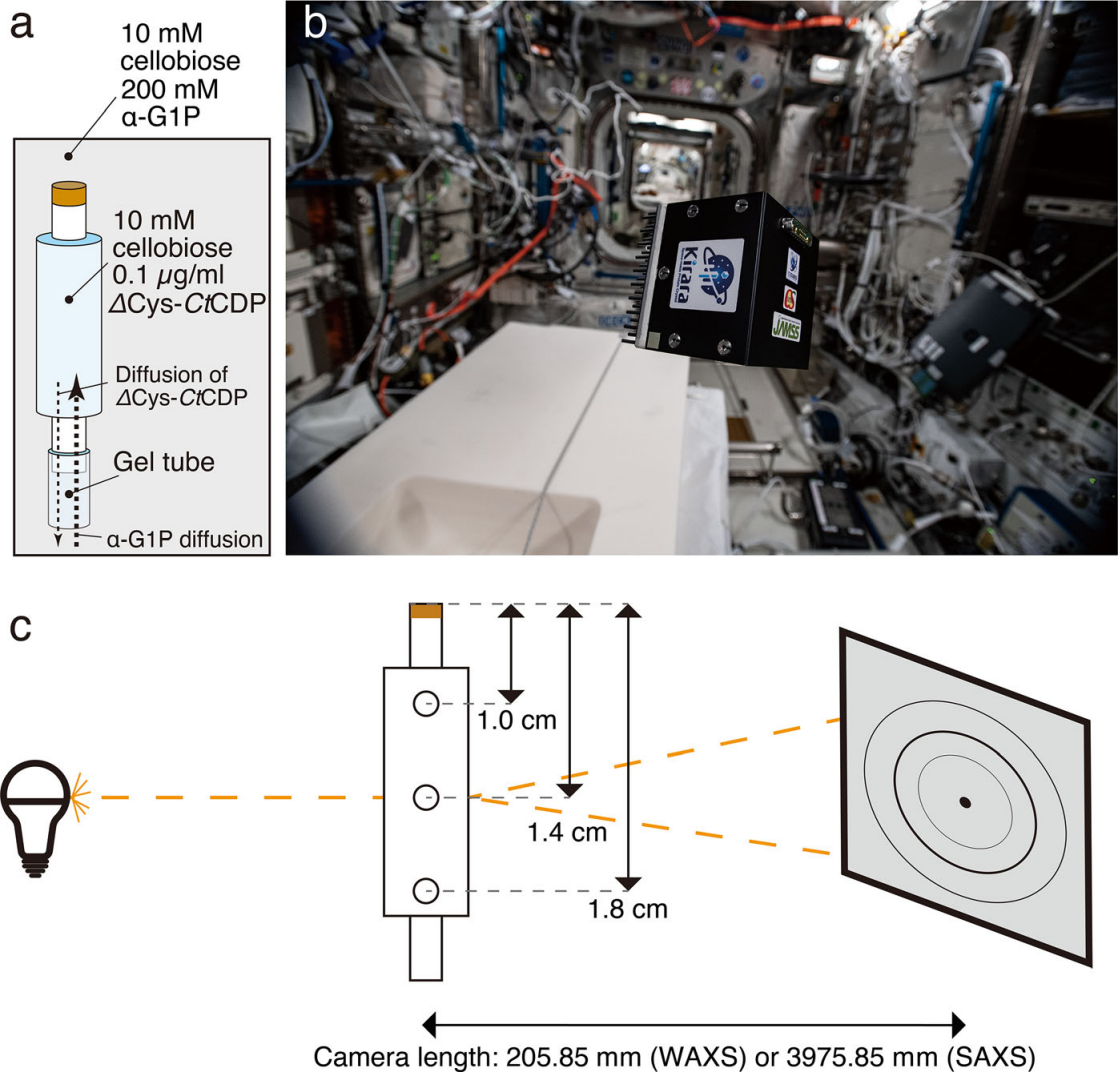
Experimental settings. **a** The counter-diffusion capillary. **b** Thermostated box containing a counter-diffusion capillary for cellulose synthesis under a microgravity environment (©NAS/ESA, Photo was from https://www.esa.int/ESA_Multimedia/Images/2021/01/ICE_Cube_commercial_COVID-19_experiment). **c** Schematic illustration of wide-angle and small-angle X-ray scattering experiment on the counter-diffusion capillary

0.10 µg/ml *Δ*cys-*Ct*CDP and 10 mM cellobiose were introduced into a C-tube counter-diffusion (Otálora et al. 2009) quartz capillary placed in 10 mM cellobiose and 200 mM α-G1P solution three days before launch. The counter-diffusion capillary consists of a 2 mm diameter quartz capillary and silicon tubing containing agarose gel; this arrangement allows the outer solution to diffuse into the capillary. Inside the counter-diffusion capillary, the initial α-G1P concentration was set to 0 mM and this gradually increased as α-G1P diffused from the gel tube (Fig. 1a). The α-G1P concentration was controlled to minimize the influence of gravity during cellulose synthesis before arrival at the ISS. The Kirara service (JAMSS, Tokyo, Japan) was used to launch the experiment to the ISS. The sample was kept in the microgravity environment of the ISS for one month at 20 ºC inside a thermostated box (Fig. 1b). The cellulose synthesized on Earth was prepared similarly, except for the presence of gravity, as a control. The capillary on the ground was set vertically so that the gravity would work to the axial direction of the capillary.

### WAXS measurements

WAXS measurements were done at the BL8S3 station of Aichi Synchrotron Radiation Center (Aichi, Japan) with a 205.85 mm camera length. Diffraction of 0.92 Å X-rays was recorded on an R-Axis IV++ (Rigaku, Tokyo, Japan), and radial integration of diffraction intensity was performed with the program FIT2D (ESRF, Grenoble, France). Sample capillaries were attached to the cell holder, and measurements were conducted at the upper part (10 mm from the capillary top), the middle part (14 mm from the capillary top), and the bottom part (18 mm from the capillary top) of the capillary (Fig. 1c).

Igor Pro (Wavemetrics, OR, US) was used to perform WAXS peak fit analysis and to create graphics. FWHM (full width at half maximum) of peaks assigned to the 020 plane of cellulose II and peak areas were determined, assuming that scattering due to water was smooth and would not form any peak.

### SAXS measurements

SAXS experiment was conducted at the BL8S3 station of Aichi Synchrotron Radiation Center under the following conditions: diffraction of 0.92 Å X-rays was recorded on an R-Axis IV++ at a camera length of 3975.85 mm. Radial integration of diffraction intensity was performed with the program FIT2D. Sample capillaries were attached to the cell holder, and measurements were done at the upper part (10 mm from the capillary top), the middle part (14 mm from the capillary top), and the bottom part (18 mm from the capillary top) of each sample capillary (Fig. 1c).

SAXS data were processed with ATSAS (Manalastas-Cantos et al. 2021) and SasView (http://www.sasview.org/). The SAXS data were analyzed after subtracting the scattering curve of the negative control solution containing 0.10 µg/ml *Δ*cys-*Ct*CDP and 10 mM cellobiose in a C-tube capillary. Earlier electron microscopy and atomic microscopy observations showed that complex structural features could co-exist in one reaction system (Hiraishi et al. 2009; Pylkkänen et al. 2020), and therefore we used a unified power law equation for fitting the data (Beaucage 1995; Tajima et al. 2019).

1 $$I\left( Q \right) = background + \mathop \sum \limits_{i = 1}^{2} \left[ {G_{i} \cdot exp\left( { - \frac{{Q^{2} \cdot Rg_{i}^{2} }}{3}} \right) + B_{i} \cdot exp\left( { - \frac{{Q^{2} \cdot Rg_{i + 1}^{2} }}{3}} \right) \cdot \left( {\frac{1}{{Q_{i}^{*} }}} \right)^{{P_{i} }} } \right]$$

2 $$Q_{i}^{*} = {\text{Q}}\left[ {erf\left( {\frac{{Q \cdot Rg_{i} }}{\sqrt 6 }} \right)} \right]^{ - 3}$$

Q, I(Q), R_g_, G, B in Eqs. (1) and (2) are scattering vector, intensity, a radius of gyration for a particular scattering body, Guinier function, and Porod-type function, respectively. The scattering vector was defined as Q = 4π/λ sinθ, where 2θ is the scattering angle, and λ is the wavelength.

### The molecular weight of enzymatically synthesized cellulose

To analyze the molecular weight and degree of polymerization of cellulose synthesized by *Δ*cys-*Ct*CDP, Matrix-assisted laser desorption/ionization time-of-flight mass spectroscopy (MALDI-ToF MS) was performed. The MALDI-ToF MS spectra were recorded on an autoflex maX (Bruker, MA, US). The solvent in capillaries containing synthesized cellulose was replaced gradually with tert-butyl alcohol. Then the cellulose samples were freeze-dried in a lyophilizer (FDU-1200, Eyela, Tokyo, Japan) and collected by breaking the capillaries with a cutting stone (Hampton Research, CA, US). Small fractions of cellulose samples were resuspended in distilled water, and the rest of the freeze-dried cellulose samples were used in SEM observation. 0.5 µl of each cellulose aqueous suspensions were mixed with 1 µl of 10 mg/ml 2,5-dihydroxybenzoic acid (Bruker, MA, US) in 50% acetonitrile-0.1% (v/v) trifluoroacetic acid, as described previously (Petrovic et al. 2015; Pylkkänen et al. 2020).

### Observation with scanning electron microscopy (SEM)

Cellulose samples were freeze-dried and collected as described in the MALDI-ToF MS section. Freeze-dried samples were coated with Pt–Pd, and SEM images were captured with an FE-SEM S-4800 (Hitachi, Tokyo, Japan) at 1 kV. Image analysis was performed on Fiji (Schindelin et al. 2012) and its plugin, DiameterJ (Hotaling et al. 2015), to determine the radii of fibrils observed in SEM observation.

## Results and discussion

### Enzyme preparation

*Ct*CDP was found to be unstable and lost its activity over several weeks. Since the synthesis of cellulose on the ISS was planned for one month, improving the stability of *Ct*CDP was the first challenge for this study. Alexander et al*.* suggested that the oxidation state of cysteine residues negatively affects the *Ct*CDP activity, and therefore, we designed *Δ*cys-*Ct*CDP in which all 11 cysteine residues are replaced with serine residues. In the present experiment, *Δ*cys-*Ct*CDP was expressed and highly purified to minimize the influence from contamination of other proteins (Supplementary Fig. 1). This *Δ*cys-*Ct*CDP did not lose activity for at least two months. Characterization of the mutated *Ct*CDP will be reported elsewhere.

### Optical observation of cellulose synthesized in counter-diffusion capillaries

**Fig. 2:**
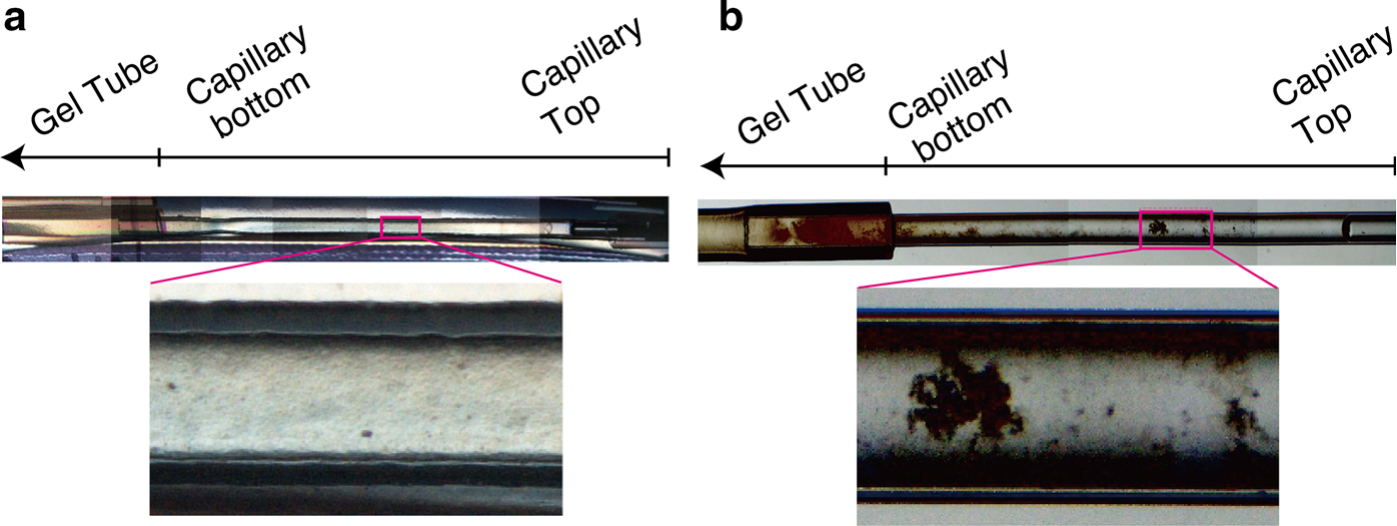
Optical observation of capillaries containing cellulose synthesized in a microgravity environment **a** and on the ground **b**. Cellulose synthesized in a microgravity environment showed no apparent aggregation, unlike cellulose synthesized on the ground

In the *Δ*cys-*Ct*CDP reaction using the counter-diffusion reaction vessel, the reaction proceeds as the donor substrate, α-G1P, is supplied from the gel tube by diffusion (Fig. 1a). In the sample capillaries, there was an unreacted region, where no product exists, on the opposite side from the gel tube. This result suggests that the enzymatic reaction proceeded sequentially from the site of the gel tube, regardless of whether the reaction takes place in space or on the ground (Fig. 2a and b). However, the appearance of the cellulose synthesized under the two conditions differed significantly.

The cellulose synthesized on the ISS had an overall homogeneous gel-like appearance, and no aggregates could be seen (Fig. 2a). However, on the ground, the formation of larger aggregates was observed, and they were more abundant near the base of the gel tube, i.e., in the direction of gravity (Fig. 2b). The density of cellulose crystals is approximately 1.6 g/cm^3^ for experimentally determined cellulose I_β_ (Daicho et al. 2019) and cellulose II theoretically determined with crystalline unit cell model (Langan et al. 2001), and under typical aqueous reaction conditions, the synthesized cellulose particles be expected to settle under gravity. This settling would not occur in the microgravity environment in space, suggesting that cellulose synthesis under microgravity prevents the formation of visible highly ordered structures and aggregates, affording more homogeneous cellulose crystals. In addition, the highly ordered structure of cellulose synthesized under microgravity was sufficiently strong to withstand its weight because no aggregation was observed after the return to the Earth.

### WAXS measurements

**Fig. 3:**
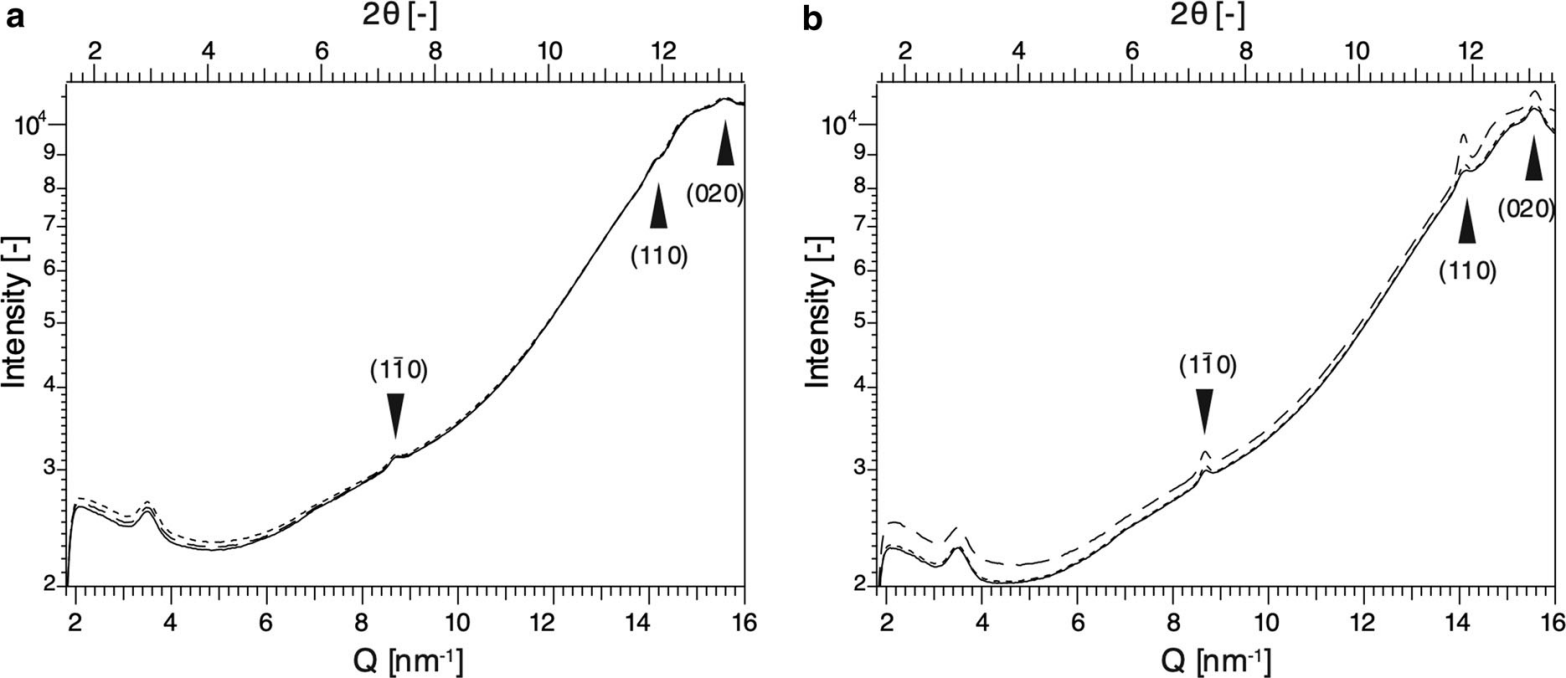
WAXS diagrams of cellulose synthesized in a microgravity environment **a** and on the ground **b**. Scattering from the upper, middle, and bottom parts are depicted by dashed, dotted, and solid lines, respectively. Cellulose synthesized in a microgravity environment had more uniform and weaker diffraction peaks of cellulose II than cellulose synthesized on the ground. Each arrow shows the location of a peak corresponding to a lattice space of cellulose II

The cellulose synthesized in space appeared homogeneous and gel-like. On the other hand, it is known that the nature of cellulose is affected by drying and other factors (Newman 2004; Hubbe et al. 2007; Kobayashi et al. 2011; Idström et al. 2013), so it was necessary to leave the cellulose in the reaction capillary to perform X-ray diffraction measurements. To identify the allomorphs of cellulose synthesized under microgravity and on the ground, WAXS diffraction measurements were conducted. The WAXS diagrams are shown in Fig. 3. The scattering intensity increased monotonically in the range of 5 nm^−1^ < Q < 16 nm^−1^ due to the presence of an excess amount of water. All measurements showed similar trends (Fig. 3). However, the scattering intensities of cellulose synthesized in space were similar along the height direction of the capillary (Fig. 3a), in contrast to the scattering intensities of cellulose synthesized on the ground, where the upper part showed higher scattering intensity in all ranges (5 nm^−1^ < Q < 16 nm^−1^, Fig. 3b). This suggests that cellulose synthesized in space has a more uniform crystal size or more uniform crystal orientation in the height direction of the capillary than cellulose synthesized on the ground.

As shown in Fig. 3, three peaks were detectable in the range of 5 nm^−1^ < Q < 16 nm^−1^. There were weak peaks in the WAXS diagram of cellulose synthesized in space, whereas cellulose synthesized on the ground showed sharp peaks. According to the formula d = 2π/Q, which describes the relationship between scattering vector (Q) and real space (d), peaks of Q = 8.69, 14.1, and 15.6 nm^−1^ correspond to real space d = 7.23, 4.45 and 4.03 Å, respectively. A combination of those d-values was matched with lattice spaces in the 1–10, 110, and 020 planes of cellulose II, respectively (Kobayashi et al. 2011; French 2014). Therefore, celluloses synthesized on the ground and under microgravity were both assigned as crystalline cellulose II. Thus, gravity did not appear to influence the polymorphic form of the product.

The areas and FWHMs of peaks attributed to the 020 plane in Fig. 3 were determined and are summarized in Table 1. The 020 plane areas of ground-synthesized cellulose II were larger than those of space-synthesized cellulose. The average peak area of cellulose on the ground was twice as large as that of cellulose synthesized in space, and the average FWHM was 10% smaller. Those data suggest that cellulose synthesized in space has a smaller crystal size or reduced degree of crystal orientation.

**Table 1:** Comparison of cellulose synthesized in space and on the ground in terms of peak areas and FWHMs derived from the 020 plane. Peak fit was performed against WAXS profiles of the upper part, middle part, and bottom part of each capillary. Peak areas derived from the 020 plane of cellulose synthesized in space were relatively small, and FWHMs were rather large compared to those of cellulose synthesized on the ground. The unit of area is arbitrary

	Cellulose synthesized in space				Cellulose synthesized on the ground			
	Upper part	Middle part	Bottom part	Average	Upper part	Middle part	Bottom part	Average
Area	125 ± 34	88.8 ± 11.7	93.9 ± 13.9	103	188 ± 8	234 ± 14	246 ± 20	222
FWHM (º)	0.379 ± 0.055	0.282 ± 0.025	0.296 ± 0.029	0.319	0.250 ± 0.008	0.292 ± 0.011	0.316 ± 0.015	0.286

### SAXS measurements

WAXS measurements confirmed that the cellulose synthesized in space was not an amorphous gel, but consisted of particles of crystalline cellulose. Therefore, SAXS measurements were carried out to obtain information on this particulate cellulose.

**Fig. 4:**
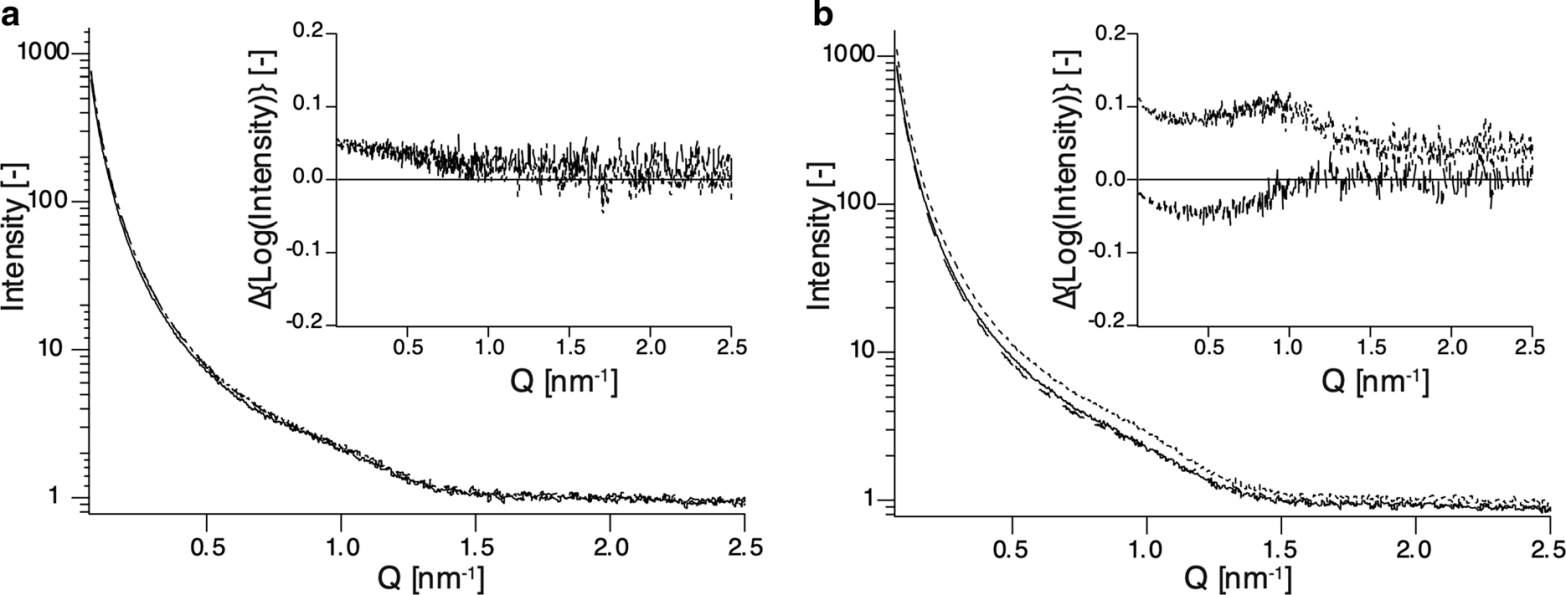
Experimental SAXS curves for *Ct*CDP-cellulose and scattering differences in the height direction of the capillaries. SAXS profiles of cellulose synthesized in space and on the ground are shown in **a** and **b**, respectively. The insets show residual scattering after subtraction of the scattering from the bottom part of capillaries. Scattering from the upper, middle, and bottom parts are depicted by dashed, dotted, and solid lines, respectively

Figure 4 shows Log-Absolute SAXS plots and residual plots after subtraction of scattering from the bottom part of each capillary. The residual plots indicate that cellulose synthesized in space showed a more uniform density of particles (Fig. 4a inset) with various radii of gyration throughout the capillary than cellulose synthesized on the ground (Fig. 4b inset). Specifically, there were more components in the region of Q < 1.0 nm^−1^ in the upper and middle parts compared to the bottom part, though there was no significant difference between the plots of the upper and middle parts. Thus, the SAXS profile of cellulose synthesized on the ground differed more depending on the position in the capillary, and the difference was particularly pronounced in the region of Q < 1.5 nm^−1^, which means the particle region with a radius of gyration R_g_ greater than 4.18 nm (Fig. 4b inset). As for cellulose synthesized on the ground, scattering from the middle part of the capillary was higher than scattering from other parts (Fig. 4b). Considering that gravitational settling is the main cause of the variation in particle distribution with capillary position, the observation of a higher density in the middle part of the capillary seems strange. However, it might be explained by adsorption of cellulose II lamellar crystals on the quartz glass during sedimentation and aggregation. *Ct*CDP-cellulose II lamellar crystals have a large hydrophilic area with abundant hydroxyl groups on the surface (Hiraishi et al. 2009; Wada et al. 2021), and might therefore bind readily with SiO_2_ at the surface of the capillary.

**Fig. 5:**
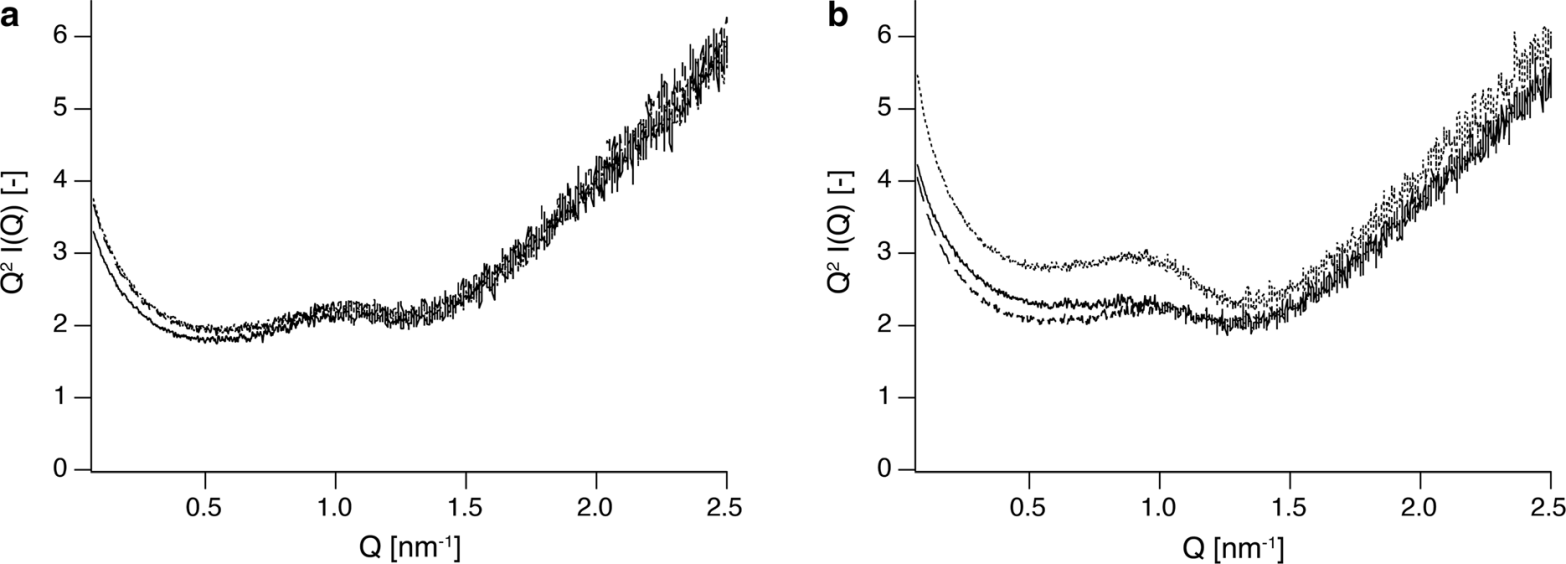
A Kratky plot demonstrating the difference in *Ct*CDP-cellulose particle distribution in the height direction of the capillaries. Kratky plot of cellulose synthesized in a microgravity environment **a** and on the ground **b**. Scattering from the upper, middle, and bottom parts is depicted by dashed, dotted, and solid lines, respectively

To further highlight the differences, a Kratky plot was performed (Fig. 5). The scattering from the center of the ground-synthesized cellulose (Fig. 5b) showed a clear peak at Q ≈ 0.90 nm^−1^, which is distinctly different from that of space-synthesized cellulose (Fig. 5a). While the scattering intensity of cellulose synthesized on the ground varied with the height in the capillary (Fig. 5b), the scattering intensity of cellulose synthesized under microgravity was relatively homogeneous (Fig. 5a). These data qualitatively suggest that there was no significant difference in the number and volume of cellulose particle scatterers in the upper or bottom part of the capillary between the ground and space conditions. Nevertheless, there was a significant difference in the number and volume of scatterers in the middle part.

**Fig. 6:**
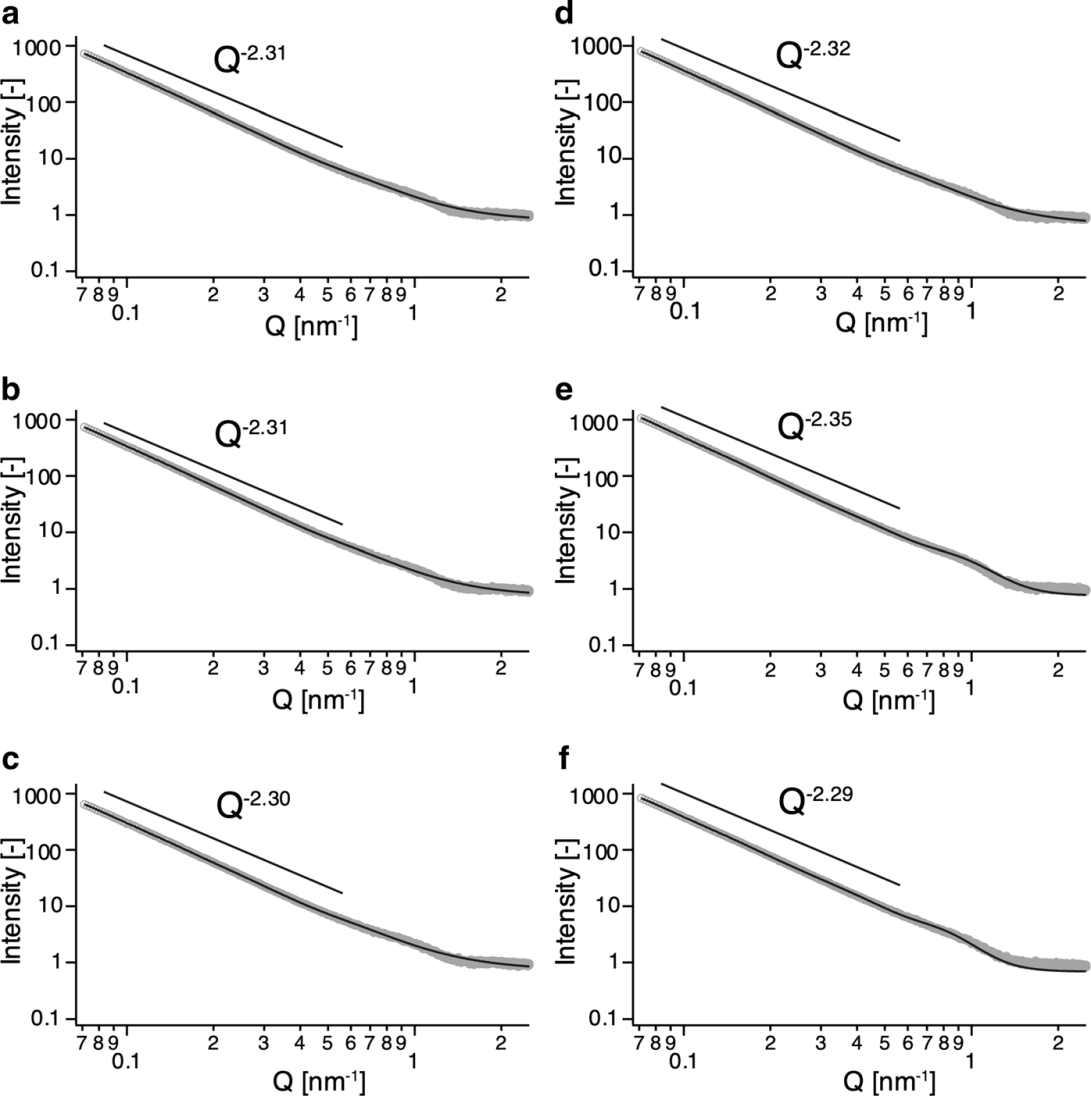
Experimental SAXS profiles of *Ct*CDP-cellulose and fitting analysis with Eq. (1). Scattering of cellulose synthesized in space from the upper part **a**, middle part **b**, and the bottom part **c** and scattering of cellulose on the ground from the upper part **d**, middle part **e**, and the bottom part **f** are depicted in log–log plots. Fitted curves are shown as solid lines, and measured values are shown as gray circles in each figure. All SAXS data in the region 0.07 nm^−1^ < Q < 0.5 nm^−1^ are proportional to approximately Q^−2.3^, indicating that the cellulose particles have a platelet shape

To quantitatively evaluate the size of the scatterers, we focused on the small-angle results in the SAXS measurements. We found that a unified power law equation (Beaucage 1995) gave a good fit, with sufficiently small values of chi^2^/point for all parts of the capillaries (Fig. 6). Especially in the region of 0.07 nm^−1^ < Q < 0.5 nm^−1^, all SAXS scatterings were proportional to Q^−2.28^ – Q^−2.35^, indicating that the particles have a thin plate shape, whether the cellulose is synthesized in space or on the ground (Kratky and Porod 1949; Pedersen 1997). This conclusion is consistent with previous studies showing that *Ct*CDP-cellulose single crystals have a platelet shape (Hiraishi et al. 2009; Pylkkänen et al. 2020; Wada et al. 2021).

The small-angle region of the SAXS results did not show a good fit in the Guinier plot analysis for all the samples. This suggests that all the samples obtained consist of a set of aggregates with multiple radii of gyration. Therefore, in this fitting analysis, we focused only on the radius of gyration R_g2_, which corresponds to the peak at Q ≈ 0.9 nm^−1^. Table 2 shows all the parameters of the fitting analysis; the average R_g2_ values for cellulose in space and on the ground were calculated to be 6.61 ± 0.09 nm and 4.57 ± 0.84 nm, respectively. It has been shown that cellulose synthesized in vitro by *Ct*CDP under batch conditions on the ground has a degree of polymerization of 9 and forms plate-like crystals with a thickness of about 5 nm (Hiraishi et al. 2009). This value is similar to the R_g2_ values of cellulose in space and on the ground. Thus, *Δ*cys-*Ct*CDP-cellulose's  crystalline lamellar structure existed in celluloses synthesized both in space and on the ground.

**Table 2:** Parameters for fitting SAXS profiles to Eq. (1). Fitting analysis was performed for SAXS profiles of the upper part, middle part, and bottom part of each capillary

	Cellulose synthesized in space			Cellulose synthesized on the ground		
	Upper part	Middle part	Bottom part	Upper part	Middle part	Bottom part
Chi^2^/points	0.0849	0.0559	0.0525	0.0492	0.195	0.140
background	0.791 ± 0.066	0.746 ± 0.065	0.735 ± 0.066	0.681 ± 0.068	0.763 ± 0.070	0.688 ± 0.062
R_g1_ (nm)	34.4 ± 0.7	35.5 ± 0.7	34.4 ± 0.8	33.5 ± 0.5	32.7 ± 0.2	32.4 ± 0.2
P_1_	2.78 ± 0.032	2.75 ± 0.03	2.80 ± 0.04	2.76 ± 0.03	2.66 ± 0.01	2.68 ± 0.01
B_1_	0.619 ± 0.051	0.67 ± 0.05	0.526 ± 0.056	0.721 ± 0.058	1.23 ± 0.02	0.943 ± 0.021
G_1_	3360 ± 125	3670 ± 151	2980 ± 134	3500 ± 100	4700 ± 53	3500 ± 55
R_g2_ (nm)	6.48 ± 0.41	6.56 ± 0.41	6.78 ± 0.45	6.22 ± 0.44	3.50 ± 0.00	4.00 ± 0.00
P_2_	2.71 ± 0.16	2.72 ± 0.15	2.61 ± 0.14	2.81 ± 0.18	5.80 ± 0.64	6.39 ± 0.73
B_2_	1.32 ± 0.10	1.34 ± 0.10	1.27 ± 0.10	1.42 ± 0.10	4.18 ± 0.32	1.94 ± 0.09
G_2_	56.4 ± 9.1	58.8 ± 9.3	58.2 ± 9.9	55.9 ± 9.8	20.7 ± 0.3	23.9 ± 0.4

The parameter B_2_ in Table 2 represents the number or density of particles having a radius of gyration R_g2_. Cellulose synthesized in space had uniform B_2_ values at all measured points (1.32, 1.34, and 1.27 for the capillary’s upper, middle, and bottom parts, respectively). In contrast, cellulose on the ground had different values (1.42, 4.18, and 1.94 for the upper, middle, and bottom parts of the capillary). This difference suggested that cellulose synthesized in space has a quantitatively more uniform density of particles with a radius of gyration R_g2_ in the height direction of capillary, as compared with cellulose on the ground.

### Analysis of molecular weight and degree of polymerization of enzymatically synthesized cellulose

**Fig. 7:**
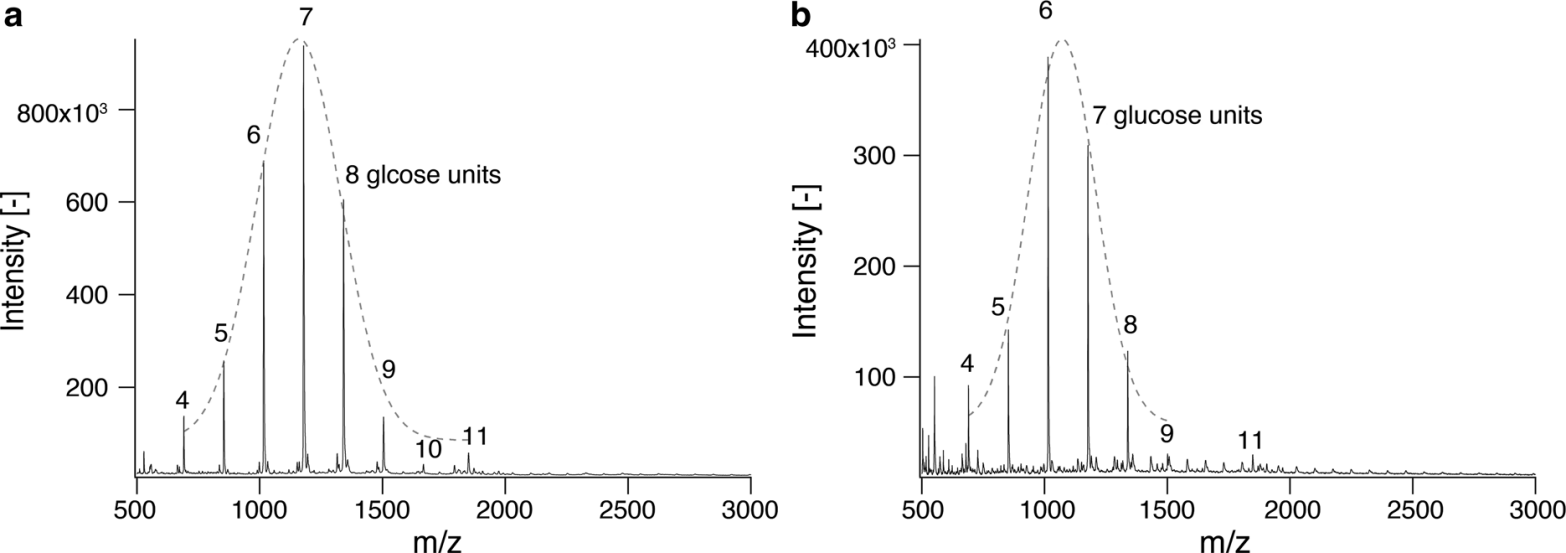
MALDI-ToF MS spectra of cellulose synthesized under microgravity **a** and on the ground **b**. Individual cellulose chains with peak-to-peak mass differences of 162 Da are indicated in terms of glucose units of molecular chains. The top peaks were approximate with Gaussian curves depicted as dotted lines in **a** and **b**

Results from SAXS showed that cellulose synthesized under microgravity had slightly larger R_g2_ values, which assumingly represented crystalline thickness and cellulose chain length. To determine molecular weight and validate SAXS results, MALDI-ToF MS was performed. In the MALDI-ToF MS spectra shown in Fig. 7, several peaks representing individual glucan chains in the range of 500–2000 Da, indicating the degree of polymerization (DP) of 4–11, were obtained. Their peak-to-peak mass differences were 162 Da, corresponding to a single glucose unit. Peak tops were approximate with the Gaussian curve, and mean molecular weights were 1160 (DP = 6.89) under microgravity and 1072 (DP = 6.35) on the ground.

Previously synthesized cellulose with initial 10 mM cellobiose and 200 mM α-G1P in a test tube had the strongest peak of DP = 7 with MALDI-ToF MS (Petrovic et al. 2015); however, cellulose synthesized on the ground had the strongest peak of DP = 6. Previous studies of *Ct*CDP-cellulose indicated that DP of the product was affected by the initial ratio of cellobiose and α-G1P: the relatively larger cellobiose concentration to α-G1P gave the smaller DP of the product. In a counter-diffusion capillary and outer solution, the initial α-G1P concentrations were set to 0 mM and 200 mM, respectively, therefore the final concentration of α-G1P in a capillary and exterior solution would be smaller than 200 mM (Petrovic et al. 2015; Pylkkänen et al. 2020). Therefore, the composition of the reaction mixture and apparatus for the reaction are the reasons to have a relatively small cellulose chain on the ground. On the other hand, under microgravity, synthesized cellulose had the strongest peak of DP = 7, which was slightly larger than cellulose synthesized on the ground, and cellulose having a maximum DP of 11 was detected (Fig. 7a). Protein and CaCO_3_ crystalline nucleation rates were reportedly several times lower under microgravity than normal gravity due to suppressed convection flow driven by the difference of solute concentration in space (Liu et al. 2000; Suzuki et al. 2019). Cellulose crystallization under microgravity would have a similar nucleation behavior, leading to a relatively long reaction time and a slightly longer cellulose chain synthesized.

According to the Stokes–Einstein equation and Fick’s law of diffusion, which explains the molecular diffusion in solution, the gravity does not affect the diffusion rate; therefore, the α-G1P concentration at every point in capillaries was considered to be the same value. The *Δ*cys-*Ct*CDP’s reaction rate had a constant relationship with the substrates’ concentration: in this situation, cellodextrins and α-G1P. From those facts, it is highly possible that the difference of cellulose DP derived not from the difference in the reaction rate of *Δ*cys-*Ct*CDP driven by the rate of α-G1P supply but the increasing reaction time of *Δ*cys-*Ct*CDP due to suppression of convection flow.

Results from MALDI-ToF MS analysis matched well with fitting analysis in SAXS experiments: the thickness of ribbons was larger under microgravity than on the ground. However, the exact values from fitting analysis in SAXS experiments were larger than results obtained from MALDI-ToF MS, considering each glucose unit has a 0.5 nm length. Under microgravity, a small fraction of cellulose having DP = 11 was detected with MALDI-ToF MS. Therefore, the difference between SAXS fitting analysis and MALDI-ToF MS was partly because SAXS fitting analysis was affected by the small fraction of long cellulose chains.

### Observation with SEM and quantification of ribbon-width

**Fig. 8:**
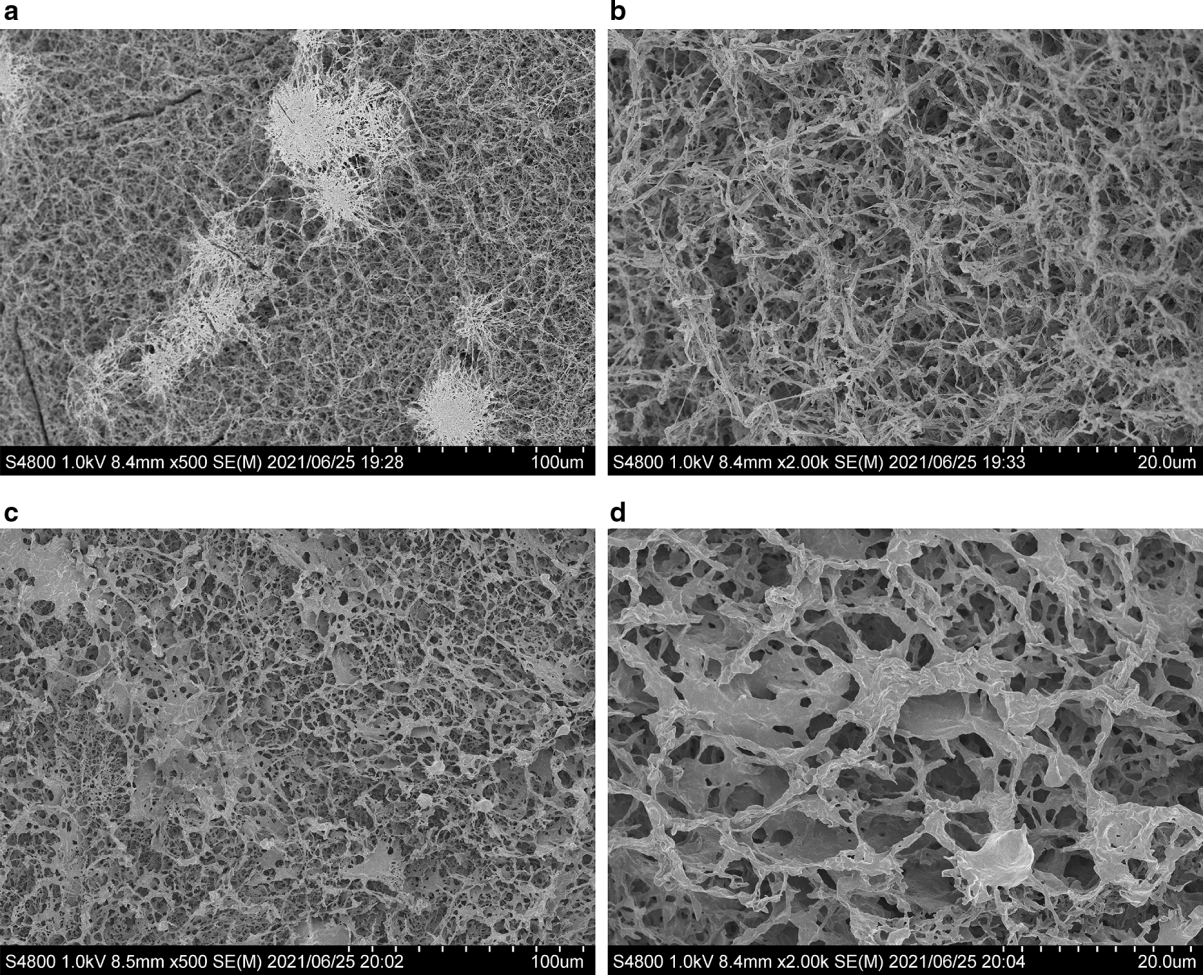
Typical SEM images of cellulose synthesized in space and on the ground. Images of cellulose synthesized in space were captured at × 500 **a** and × 2000 **b** magnification. **c** and **d** show images of cellulose synthesized on the ground at × 500 and × 2000 magnification, respectively. Cellulose synthesized under a microgravity environment generated a network consisting of thinner ribbons, while cellulose synthesized on the ground had a network structure with matrix-like thick ribbons

Typical SEM images are shown in Fig. 8. Cellulose synthesized in space (Fig. 8a and b) had a finer network structure than cellulose synthesized on the ground (Fig. 8c and d). The network consisted of ribbon-like structures estimated to be several hundred nm wide in space-synthesized cellulose (Fig. 8b). In contrast, cellulose synthesized on the ground contained thicker aggregates with micrometer scale width (Fig. 8d), i.e., several times larger. In Fig. 8c, the aggregations of cellulose synthesized on the ground, which lost the shape of a ribbon, were observed. Cellulose synthesized under microgravity also had an aggregate-like system (Fig. 8a), but the system maintained each ribbons’ form and behaved as a node of the network. Therefore the node of the cellulose ribbons’ network was not the result of sedimentation by gravity and might be caused by spherulites observed as previously enzymatically synthesized cellulose II on the ground (Kobayashi et al. 2000).

**Fig. 9:**
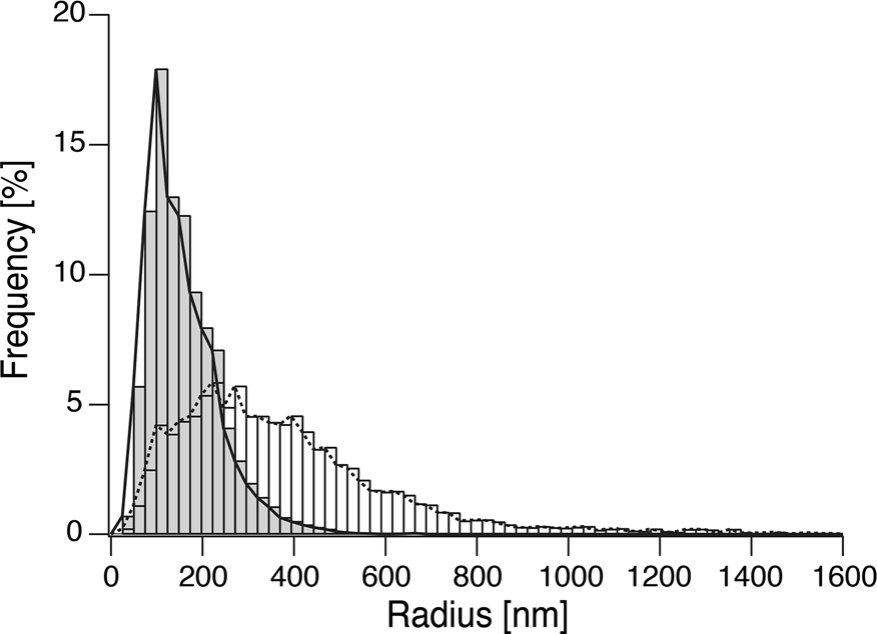
Distributions of cellulose fiber radii in the SEM images. Image analysis was performed on Fig. 8c and d with DiameterJ. The radii frequency of cellulose synthesized under microgravity was shown as gray bars and a solid line, and that of cellulose synthesized on the ground was depicted as white bars and a dotted line

To quantify the size of ribbons in network structure, image analysis was performed on Fig. 8b and d with Fiji and its plugin, DiameterJ, assuming all ribbons were cylindrical objects. From 76,869 points and 30,190 points for cellulose synthesized in space and on the ground, respectively, the frequency of radii, meaning the half-width of the ribbons, was shown in Fig. 9. Cellulose II ribbons’ mean diameter (width) were 0.254 µm and 0.584 µm for cellulose synthesized under microgravity and on the ground, respectively. The standard deviations for ribbon diameter of cellulose synthesized under microgravity and on the ground were 0.128 µm and 0.370 µm, respectively (Table. 3). A broader range of diameter was confirmed in cellulose synthesized on the ground than in space. From image analysis, cellulose synthesized under microgravity had half the mean width of the ribbons and its narrow distribution, meaning that the formation mechanism of the ribbons is completely different from cellulose synthesized on the ground.

**Table 3:** Summary of cellulose ribbon diameter determined through image analysis

	Cellulose synthesized in space	Cellulose synthesized on the ground
Diameter mean (µm)	0.254	0.584
Diameter standard deviation (µm)	0.128	0.370
Diameter mode (µm)	0.197	0.442
Diameter median (µm)	0.295	0.639
Minimum diameter (µm)	0.0491	0.0491
Maximum diameter (µm)	1.38	3.49
Skewness of distribution	1.31	1.65
Kurtosis of distribution	2.70	3.91
Number of measurements	76,869	30,190

The width of the thin ribbon-like structures synthesized in space was consistent with previous TEM and AFM observations of CDP-synthesized crystalline plate of cellulose II (Hiraishi et al. 2009; Pylkkänen et al. 2020). This indicates that the ribbon-like structure grown under microgravity was comprised of single to several cellulose crystals, while ribbons of cellulose synthesized on the ground contained more crystals. These results and the WAXS peak intensities suggest that the thick aggregated form might assemble through orientation or crystallization. It is noted that in the previous study, the thickness of ribbons to the present study only when initial cellobiose concentration and subsequent synthesized cellulose concentration were lower (Pylkkänen et al. 2020).

These partial features of the network structure are consistent with the sparse (Fig. 8a, b) and dense (Fig. 8c, d) micrometer-scale appearance of the cellulose network structure. The scale of these ribbon widths is similar to the scale of the wavelength of the visible light; therefore, these features would affect the optical appearance (Fig. 2).

It is well known that cellulose II synthesized by *Ct*CDP self-assembles into a network structure (Pylkkänen et al. 2020). Such cellulose II synthesized by *Ct*CDP on the ground was observed as white precipitates or aggregates in the earlier studies, in contrast to the gel-like appearance of space-synthesized cellulose (Fig. 2a). In previous attempts to create gel-like products with supermolecular network architecture on the ground, researchers have added nanocrystals of polymers such as polyethylene glycol and cellulose I_β_ to the reaction mixture for cellulose synthesis by *Ct*CDP (Hata et al. 2017, 2018) to serve as scaffolds. We believe the present report is the first to describe the production of pure cellulose II crystalline gel without sedimentation and aggregation.

Our observations indicate that once the cellulose II network structure is formed in space, the supermolecular structure is stable after return to Earth. A relatively light polymer (PMMA, 1.2 g/cm^3^) was reported to form a network structure of crystalline polymer through viscoelastic phase separation on the ground, and gravity appeared to have a negligible influence for at least 12 h (Tsurusawa et al. 2017). Thus, the effect of gravity on cellulose may arise because of the high specific gravity of cellulose compared with water. Therefore, the microgravity environment in space may be essential for the production of cellulose II crystalline gel.

## Conclusion

In the present study, we investigated the possibility that gravity influences the crystallization and formation of the highly ordered structure of cellulose II. We found that cellulose synthesized in space did not form aggregates like those of cellulose synthesized on the ground. WAXS demonstrated that similar nano-scale crystalline cellulose II packing occurred on the ground and in space. However, the SAXS experiment showed that cellulose particles in a capillary had higher homogeneity when synthesized in space. SEM observation showed that space-synthesized cellulose had a fine supramolecular network structure on the micrometer scale, and this was strong enough to survive after return to Earth. These findings suggest that gravity influences aggregate formation during self-assembly to form the network. In this work, a bottom-up synthesis of pure cellulose II crystal gel without sedimentation and aggregation was achieved for the first time. The physical properties of this newly created cellulose II crystalline gel remain to be investigated.

## Supplementary Information

Below is the link to the electronic supplementary material.

Supplementary file1 (DOCX 956 kb)

## References

[CR1] Alexander JK (1968). Purification and specificity of cellobiose phosphorylase from *Clostridium thermocellum*. J Biol Chem.

[CR2] Atalla RH, VanderHart DL (1984). Native cellulose: a composite of two distinct crystalline forms. Science.

[CR3] Beaucage G (1995). Approximations leading to a unified exponential/power-law approach to small-angle scattering. J Appl Crystallogr.

[CR4] Belton PS, Tanner SF, Cartier N, Chanzy H (1989). High-resolution solid-state ^13^C nuclear magnetic resonance spectroscopy of tunicin, an animal cellulose. Macromolecules.

[CR5] Brown RM (1996). The biosynthesis of cellulose. J Macromol Sci - Pure Appl Chem.

[CR6] Daicho K, Kobayashi K, Fujisawa S, Saito T (2019). Crystallinity-independent yet modification-dependent true density of nanocellulose. Biomacromol.

[CR7] Fontana P, Schefer J, Pettit D (2011). Characterization of sodium chloride crystals grown in microgravity. J Cryst Growth.

[CR8] French AD (2014). Idealized powder diffraction patterns for cellulose polymorphs. Cellulose.

[CR9] Hata Y, Kojima T, Koizumi T (2017). Enzymatic synthesis of cellulose oligomer hydrogels composed of crystalline nanoribbon networks under macromolecular crowding conditions. ACS Macro Lett.

[CR10] Hata Y, Sawada T, Sakai T, Serizawa T (2018). Enzyme-catalyzed bottom-up synthesis of mechanically and physicochemically stable cellulose hydrogels for spatial immobilization of functional colloidal particles. Biomacromol.

[CR11] Hiraishi M, Igarashi K, Kimura S (2009). Synthesis of highly ordered cellulose II *in vitro* using cellodextrin phosphorylase. Carbohydr Res.

[CR12] Hotaling NA, Bharti K, Kriel H, Simon CG (2015). DiameterJ: a validated open source nanofiber diameter measurement tool. Biomaterials.

[CR13] Hubbe MA, Venditti RA, Rojas OJ (2007). What happens to cellulosic fibers during papermaking and recycling? a review. BioResources.

[CR14] Idström A, Brelid H, Nydén M, Nordstierna L (2013). CP/MAS ^13^C NMR study of pulp hornification using nanocrystalline cellulose as a model system. Carbohydr Polym.

[CR15] Inaka K, Takahashi S, Aritake K (2011). High-quality protein crystal growth of mouse lipocalin-type prostaglandin D synthase in microgravity. Cryst Growth Des.

[CR16] Inatomi Y, Sakata K, Arivanandhan M (2015). Growth of In _x_Ga_1 − x_Sb alloy semiconductor at the international space station (ISS) and comparison with terrestrial experiments. npj Microgravity.

[CR17] Kim NH, Imai T, Wada M, Sugiyama J (2006). Molecular directionality in cellulose polymorphs. Biomacromol.

[CR18] Kobayashi K, Kimura S, Togawa E, Wada M (2011). Crystal transition from cellulose II hydrate to cellulose II. Carbohydr Polym.

[CR19] Kobayashi S (2005). Challenge of synthetic cellulose. J Polym Sci Part A Polym Chem.

[CR20] Kobayashi S, Hobson LJ, Sakamoto J (2000). Formation and structure of artificial cellulose spherulites via enzymatic polymerization. Biomacromol.

[CR21] Kobayashi S, Kashiwa K, Kawasaki T, Shoda SI (1991). Novel method for polysaccharide synthesis using an enzyme: the first in vitro synthesis of cellulose via a nonbiosynthetic path utilizing cellulase as catalyst. J Am Chem Soc.

[CR22] Kobayashi S, Shoda S (1995). Chemical synthesis of cellulose and cello-oligomers using a hydrolysis enzyme as a catalyst. Int J Biol Macromol.

[CR23] Kolpak FJ, Blackwell J (1976). Determination of the structure of cellulose II. Macromolecules.

[CR24] Kratky O, Porod G (1949). Diffuse small-angle scattering of x-rays in colloid systems. J Colloid Sci.

[CR25] Krishnareddy M, Kim Y-K, Kitaoka M (2002). Cellodextrin phosphorylase from *Clostridium thermocellum* YM4 strain expressed in *Escherichia coli*. J Appl Glycosci.

[CR26] Langan P, Nishiyama Y, Chanzy H (1999). A revised structure and hydrogen-bonding system in cellulose II from a neutron fiber diffraction analysis. J Am Chem Soc.

[CR27] Langan P, Nishiyama Y, Chanzy H (2001). X-ray structure of mercerized cellulose II at 1 Å resolution. Biomacromol.

[CR28] Larsson PT, Wickholm K, Iversen T (1997). A CP/MAS ^13^C NMR investigation of molecular ordering in celluloses. Carbohydr Res.

[CR29] Liu XY, Tsukamoto K, Sorai M (2000). New kinetics of CaCO_3_ nucleation and microgravity effect. Langmuir.

[CR30] Manalastas-Cantos K, Konarev PV, Hajizadeh NR (2021). ATSAS 3.0: expanded functionality and new tools for small-angle scattering data analysis. J Appl Crystallogr.

[CR31] Nakamura A, Ishida T, Kusaka K (2015). “Newton’s cradle” proton relay with amide-imidic acid tautomerization in inverting cellulase visualized by neutron crystallography. Sci Adv.

[CR32] Nakatsubo F, Kamitakahara H, Hori M (1996). Cationic ring-opening polymerization of 3,6-di-O-benzyl-α-d-glucose 1,2,4-orthopivalate and the first chemical synthesis of cellulose. J Am Chem Soc.

[CR33] Newman RH (2004). Carbon-13 NMR evidence for cocrystallization of cellulose as a mechanism for hornification of bleached kraft pulp. Cellulose.

[CR34] Nishiyama Y, Langan P, Chanzy H (2002). Crystal structure and hydrogen-bonding system in cellulose I_β_ from synchrotron X-ray and neutron fiber diffraction. J Am Chem Soc.

[CR35] Nishiyama Y, Sugiyama J, Chanzy H, Langan P (2003). Crystal structure and hydrogen bonding system in cellulose I_α_ from synchrotron X-ray and neutron fiber diffraction. J Am Chem Soc.

[CR36] O’Neill EC, Pergolizzi G, Stevenson CEM (2017). Cellodextrin phosphorylase from *Ruminiclostridium thermocellum*: X-ray crystal structure and substrate specificity analysis. Carbohydr Res.

[CR37] Otálora F, Gavira JA, Ng JD, García-Ruiz JM (2009). Counterdiffusion methods applied to protein crystallization. Prog Biophys Mol Biol.

[CR38] Pedersen JS (1997). Analysis of small-angle scattering data from colloids and polymer solutions: modeling and least-squares fitting. Adv Colloid Interface Sci.

[CR39] Petrovic DM, Kok I, Woortman AJJ (2015). Characterization of oligocellulose synthesized by reverse phosphorolysis using different cellodextrin phosphorylases. Anal Chem.

[CR40] Pylkkänen R, Mohammadi P, Arola S (2020). *In Vitro* synthesis and self-assembly of cellulose ii nanofibrils catalyzed by the reverse reaction of *Clostridium thermocellum* cellodextrin phosphorylase. Biomacromol.

[CR41] Saxena IM, Brown RM (2005). Cellulose biosynthesis: current views and evolving concepts. Ann Bot.

[CR42] Schindelin J, Arganda-Carreras I, Frise E (2012). Fiji: an open-source platform for biological-image analysis. Nat Methods.

[CR43] Sheth K, Alexander JK (1969). Purification and properties of β-1,4-oligoglucan:orthophosphate glucosyltransferase from *Clostridium thermocellum*. J Biol Chem.

[CR44] Snell EH, Weisgerber S, Helliwell JR (1995). Improvements in lysozyme protein crystal perfection through microgravity growth. Acta Crystallogr Sect D Biol Crystallogr.

[CR45] Suzuki Y, Fujiwara T, Tsukamoto K (2019). Very low nucleation rates of glucose isomerase crystals under microgravity in the international space station. Cryst.

[CR46] Tachioka M, Nakamura A, Ishida T (2017). Production of large-volume cellulase crystals for visualization of hydrogen atoms. Int J Microgravity Sci Appl.

[CR47] Tajima H, Penttilä PA, Imai T (2019). Observation of *in vitro* cellulose synthesis by bacterial cellulose synthase with time-resolved small angle X-ray scattering. Int J Biol Macromol.

[CR48] Tanaka H, Koizumi S, Hashimoto T (2007). Self-assembly of synthetic cellulose during in-vitro enzymatic polymerization process as studied by a combined small-angle scattering method. Macromolecules.

[CR49] Tsurusawa H, Russo J, Leocmach M, Tanaka H (2017). Formation of porous crystals via viscoelastic phase separation. Nat Mater.

[CR50] Uryu T, Yamaguchi C, Morikawa K (1985). Ring-opening polymerization of 1,4-Anhydro-2,3,6-tri-O-benzyl-α-d-glucopyranose and 1,4-Anhydro-2,3,6-tri-O-benzyl-β-d-galactopyranose. Macromolecules.

[CR51] Uryu T, Yamanouchi J, Kato T (1983). Selective ring-opening polymerization of di-o-methylated and Di-O-benzylated 1,4-Anhydro-a-d-ribopyranoses and structure proof of synthetic cellulose-type polysaccharide (1→4)-β-d-ribopyranan and (1→5)-α-d-Ribofuranan. J Am Chem Soc.

[CR52] VanderHart DL, Atalla RH (1984). Studies of microstructure in native celluloses using solid-state ^13^C NMR. Macromolecules.

[CR53] Vekilov PG (1999). Protein crystal growth - microgravity aspects. Adv Sp Res.

[CR54] Wada M, Wakiya S, Kobayashi K (2021). Three-dimensional alignment of cellulose II microcrystals under a strong magnetic field. Cellulose.

[CR55] Yamaguchi S, Sunagawa N, Matsuyama K (2021). Preparation of large-volume crystal of cellulase under microgravity to investigate the mechanism of thermal stabilization. Int J Microgravity Sci Appl.

