# Supplementary material for: Enzymatic synthesis of cellulose in space: gravity is a crucial factor for building cellulose II gel structure
Source: Cellulose (Lond). 2022 Jan 29;29(5):2999–3015. doi: 10.1007/s10570-021-04399-0 (PMC8800430; doi:10.1007/s10570-021-04399-0)
Supplement: Supplementary file 1 — Supplementary file1 (DOCX 956 kb) [file 10570_2021_4399_MOESM1_ESM.docx]

Supplementary Materials for

**Enzymatic synthesis of cellulose in space: gravity is a crucial factor for building cellulose II gel structure**

Tomohiro Kuga^1^, Naoki Sunagawa^1^ and Kiyohiko Igarashi^1,2*^


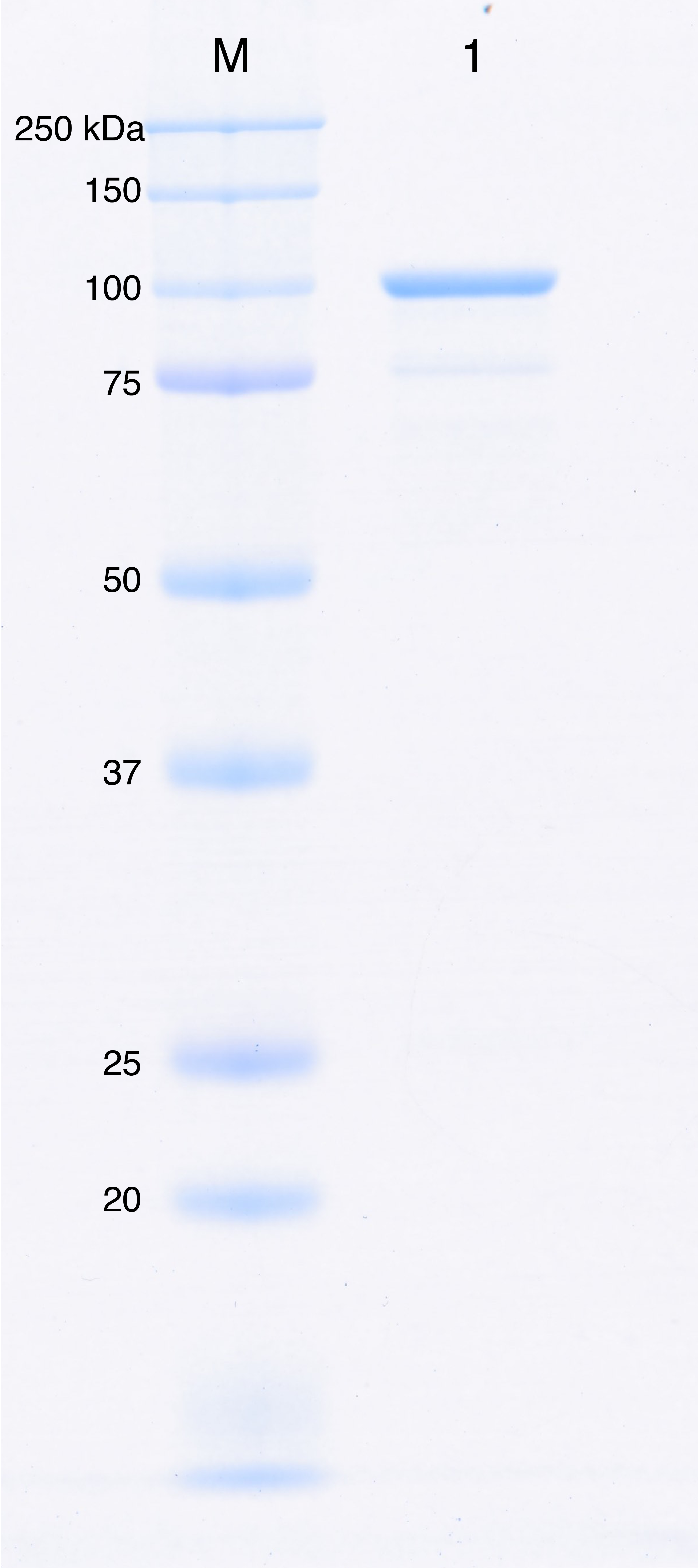


Supplementary Fig. 1. SDS-PAGE of *Δ*cys-CtCDP. Lane M, molecular weight marker; Lane 1, *Δ*cys-*Ct*CDP after two-step purifications.
